# Endoscopic Resection for Iliopectineal Bursitis Associated With Developmental Dysplasia of the Hip

**DOI:** 10.7759/cureus.23515

**Published:** 2022-03-26

**Authors:** Masashi Fujii, Hiroaki Kijima, Mitsunori Kaya, Naohisa Miyakoshi

**Affiliations:** 1 Orthopedic Surgery, Akita City Hospital, Akita, JPN; 2 Orthopedic Surgery, Akita University Graduate School of Medicine, Akita, JPN; 3 Orthopedic Surgery, Kaya Orthopedic Sports Clinic, Sapporo, JPN

**Keywords:** extra-articular pathology of the hip, hip endoscopy, hip arthroscopy, developmental dysplasia of the hip, iliopectineal bursitis

## Abstract

Iliopectineal bursitis usually develops subsequent to other hip pathologies and can often be treated conservatively. However, when conservative treatment fails or the enlarged bursa causes pain or compression of the surrounding neurovascular structures, surgery may be required. Most previous studies have described open surgeries, and reports on endoscopy are very limited. We present a case of iliopectineal bursitis associated with developmental dysplasia of the hip (DDH) that was successfully treated endoscopically. A 16-year-old female with a one-year history of right inguinal pain was referred to our department. She was diagnosed with a hip ganglion and treated with needle aspiration nine times by her previous doctor. Radiographs revealed bilateral DDH without narrowing of the joint space. Magnetic resonance imaging revealed a distinct mass in the deep layer of the iliopsoas muscle, and communication between the mass and the hip joint was observed on ultrasonography. Endoscopic debridement and resection were performed based on the diagnosis of iliopectineal bursitis. We partially debrided the medial side of the rectus femoris muscle toward the deep layer and resected the bursa. We observed a burst of concentrated content from the bursa and confirmed the disappearance of the mass by intraoperative ultrasonography. The postoperative course was good, and there were no functional restrictions or symptom recurrence at two-year postoperatively. Endoscopic resection for repetitive iliopectineal bursitis without an intraarticular procedure does not induce hip instability in patients with DDH and is a minimally invasive cosmetic procedure, and superior to open surgery, especially in young women.

## Introduction

The iliopectineal bursa is the largest bursa in the human body and is present in 98% of adults [1]. Owing to its location between the iliopsoas muscle and capsule, bursitis is not uncommon and can frequently lead to anterior hip pain. It usually develops after other hip pathologies, making primary bursitis relatively rare [2]. Most patients can be treated conservatively with good outcomes. However, invasive management is sometimes necessary. Almost all studies on surgical intervention for iliopectineal bursitis have been performed using open surgeries [3-5]. Studies of arthroscopic and endoscopic treatment are limited, and there is no consensus on surgical treatment strategies for this pathology associated with developmental dysplasia of the hip (DDH). Herein, we report a case of repetitive iliopectineal bursitis associated with DDH in a young female patient, which required surgery, as well as the successful results of the treatment via endoscopy.

## Case presentation

**Figure 1:**
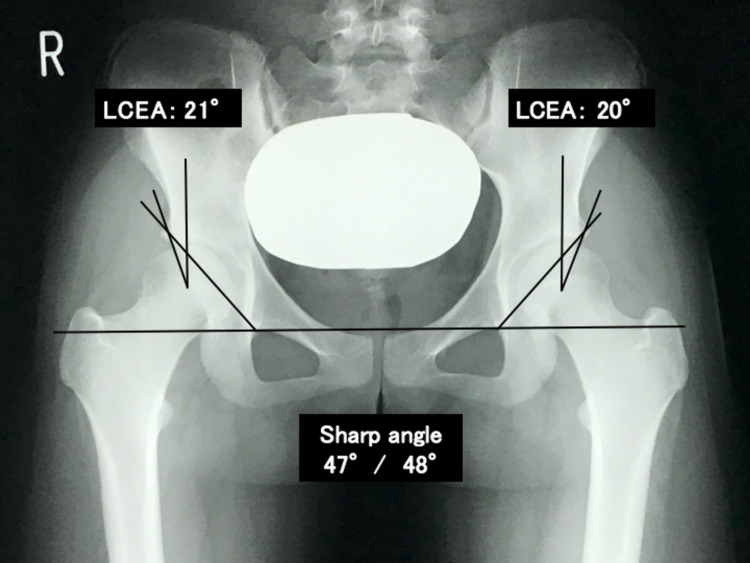
Radiograph, anteroposterior view, shows no findings of osteoarthritis. LCEA: lateral center-edge angles

**Figure 2:**
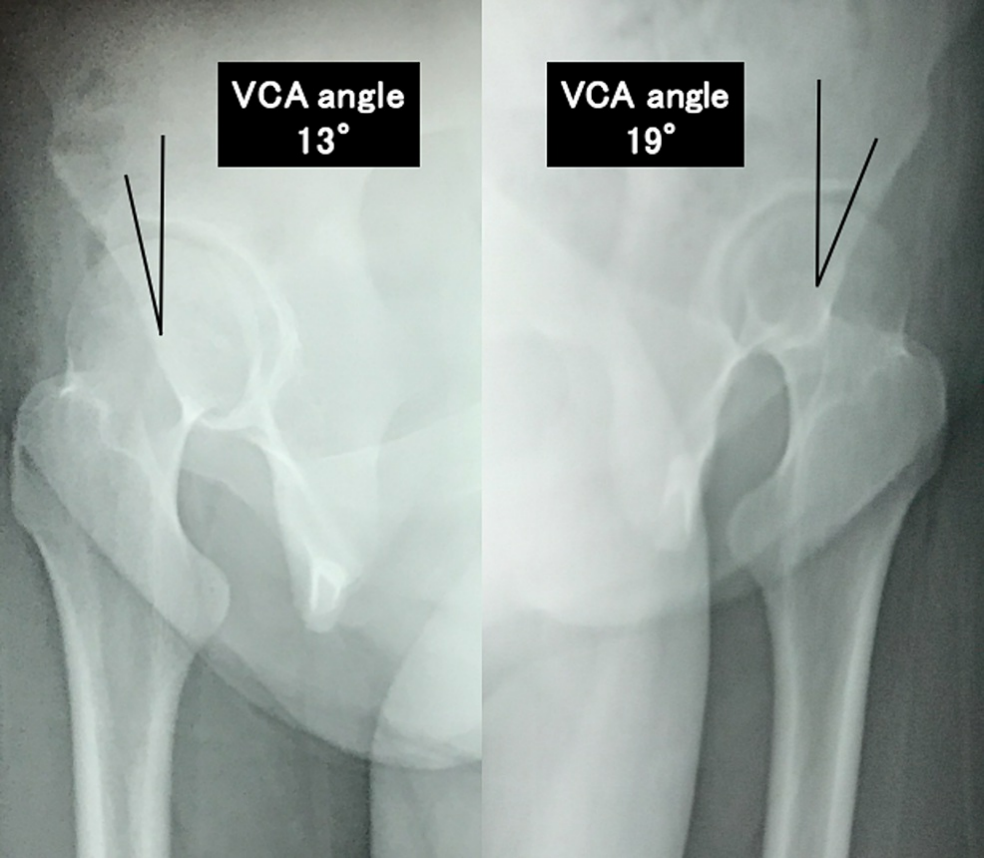
Radiograph, false profile view, shows bilateral acetabular dysplasia of the hip. VCA: vertical center anterior

A 16-year-old female presented with a one-year history of right-sided inguinal pain. She was diagnosed with a hip ganglion and treated with needle aspiration nine times by her previous doctor. The patient was referred to our department because of repeated recurrences. She had severe pain in the anterior hip and a globally restricted range of motion. Her modified Harris hip score (mHHS) was 60.5. Radiography revealed bilateral DDH without joint space narrowing. The lateral center-edge angles (LCEA) were 21° and 20°, Sharp’s angles were 47° and 48° (Figure 1), and the vertical center anterior (VCA) angles on the false profile view were 13° and 19° (Figure 2).

**Figure 3:**
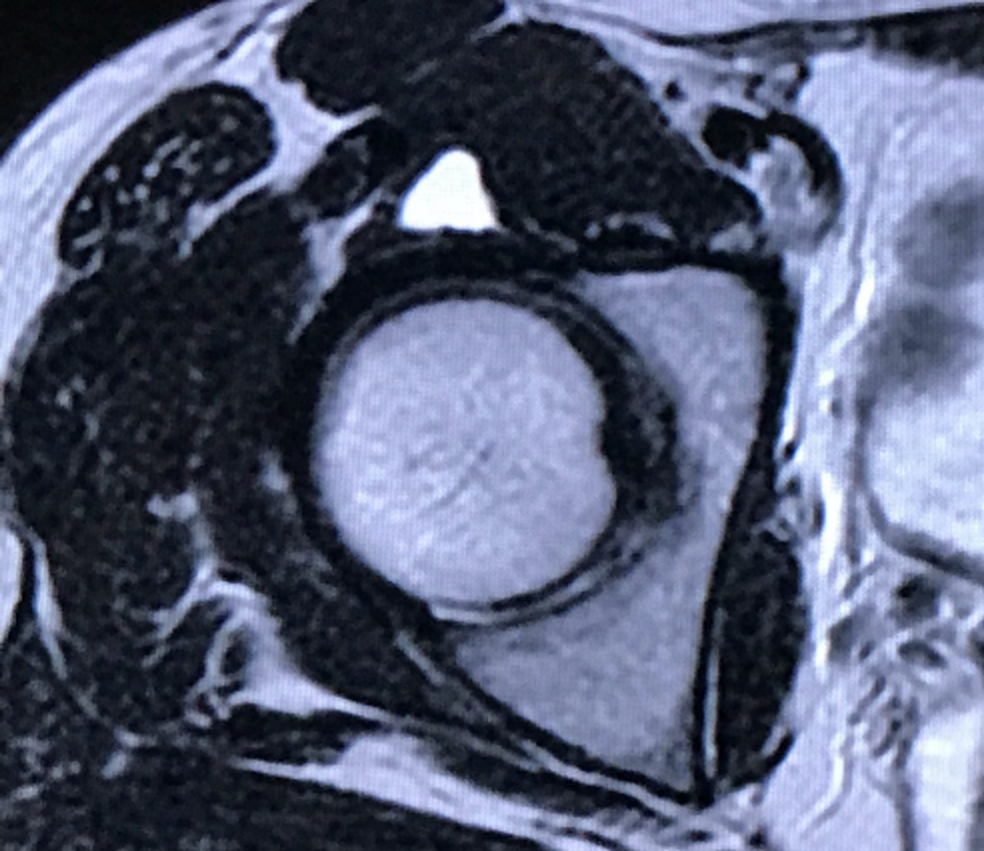
Preoperative MRI, axial view, shows the high-intensity mass between the iliopsoas muscle and anterior capsule.

**Figure 4:**
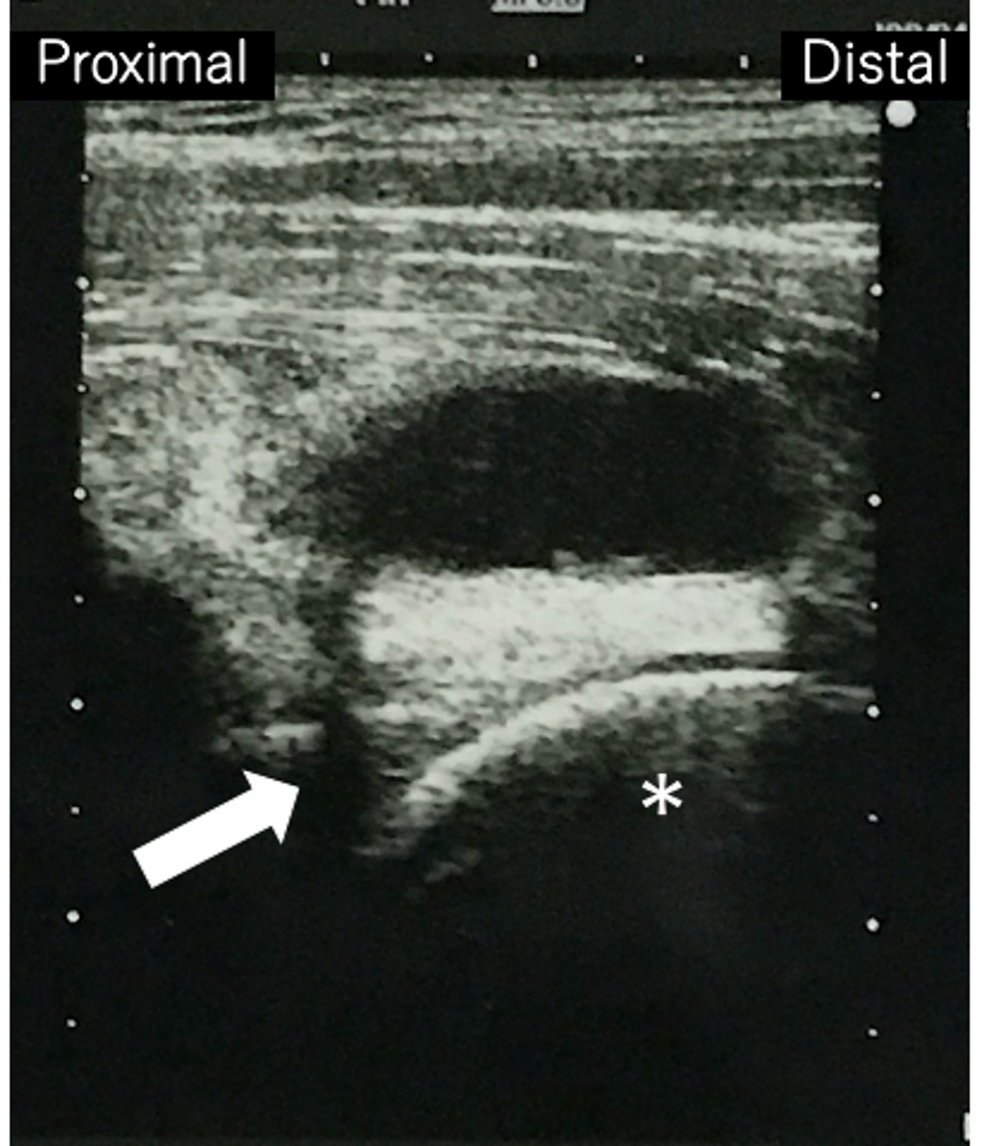
Preoperative ultrasonography image shows the hypoechoic mass anterior to the femoral head (asterisk) and its communication with the intraarticular space (arrow).

All measurements were reported for the right side, followed by the left side. T2-weighted magnetic resonance imaging revealed a clear, distinct mass in the deep layer of the iliopsoas muscle (Figure 3). Ultrasonography revealed a hypoechoic mass over the femoral head that communicated with the hip joint (Figure 4).

**Figure 5:**
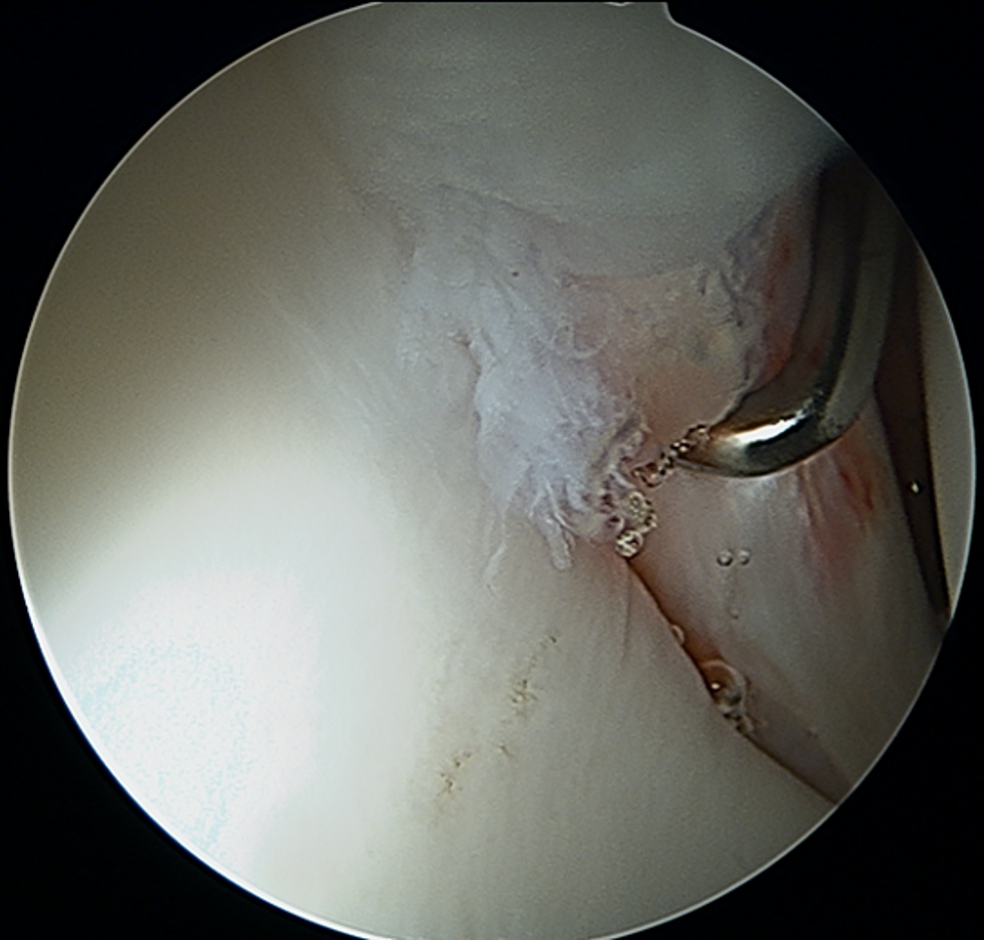
Intraoperative photograph of the intraarticular space shows minimally flayed labrum.

Power Doppler assessment revealed no evidence of increased blood flow. We arrived at a diagnosis of iliopectineal bursitis and performed aspiration and steroid injection. The puncture fluid was light yellow and serous, and immediately after the puncture, the symptoms disappeared completely; however, both the mass and symptoms recurred within two weeks. Therefore, bursa resection was indicated, and an endoscopic procedure was planned. The operation was performed under general and epidural anesthesia, traction of the lower limb in the supine position. First, the intraarticular space was observed using the conventional anterolateral portal (ALP) and mid-anterior portal (MAP). Considering joint instability due to DDH, we did not cut the capsule and only observed the intraarticular space. We confirmed a minimally flayed labrum and the absence of abnormal synovial hyperplasia, free bodies, and labral tearing, which causes synovial fluid leakage (Figure 5). Subsequently, we released the traction and moved the exterior of the endoscopes to the capsule and expanded the extraarticular space. We then observed the relief of the rectus femoris tendon and its origin on the anterior inferior iliac spine (AIIS). There were no signs of inflammation or tendinosis around the rectus femoris tendon.

**Figure 6:**
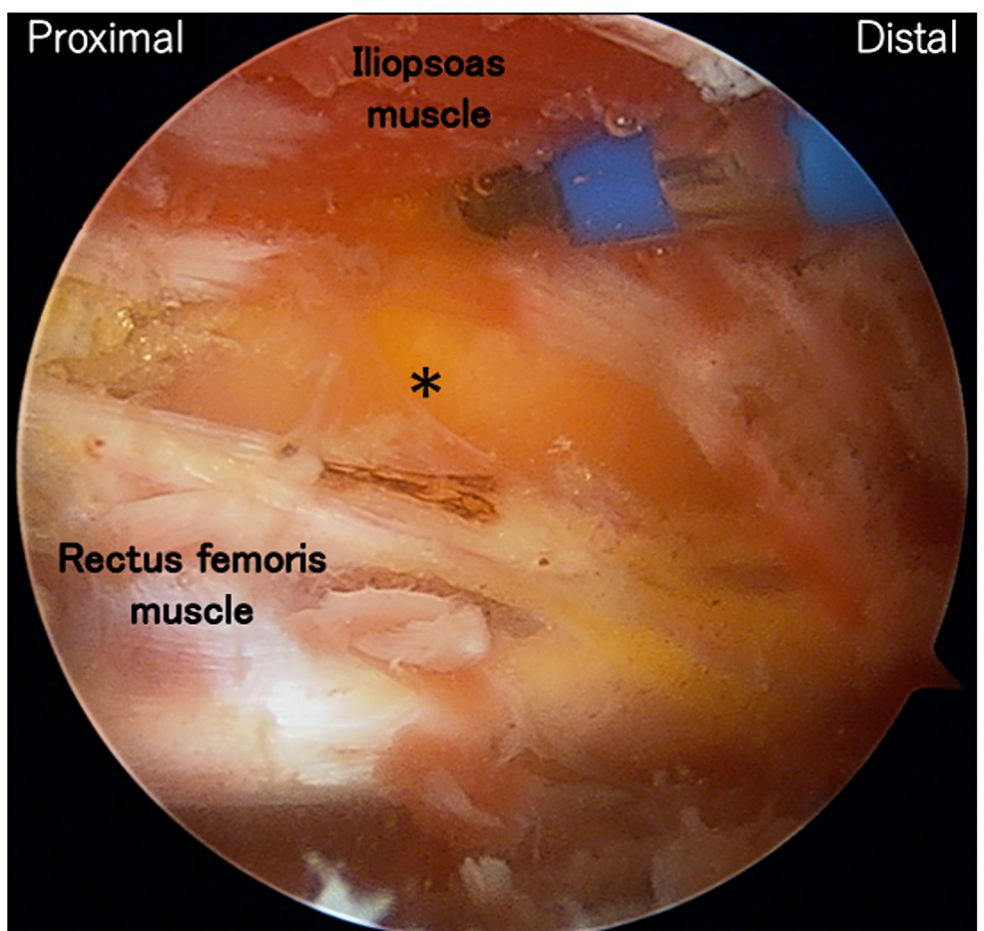
Intraoperative photograph of the extraarticular space shows the bursa is burst by debriding the space between the rectus femoris and the iliopsoas muscles (asterisk).

We also debrided the medial side of the rectus femoris muscle toward the deep layer of the iliopsoas muscle via the MAP and observed a burst of slightly concentrated yellow content from the bursa upon rupture (Figure 6). In addition, we debrided the space between the deep layer of the iliopsoas muscle and the surface of the capsule. We also confirmed the resolution of the mass using intraoperative ultrasonography.

**Figure 7:**
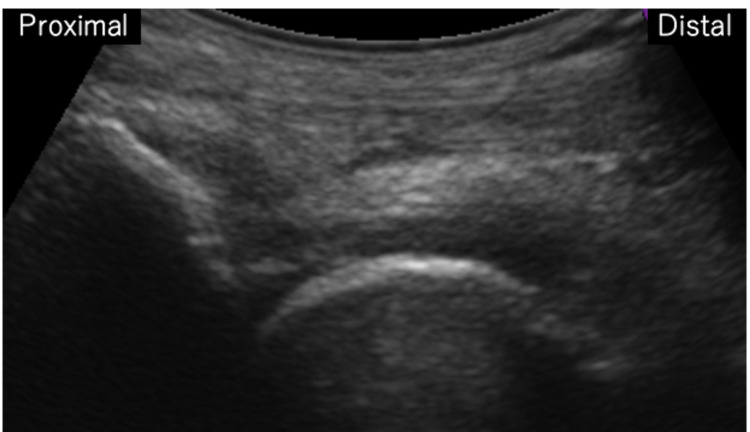
Two-year postoperative follow-up ultrasonography shows the mass in front of the hip joint is disappeared.

All procedures finished in two hours and there were no problems with no nerves appearing in the surgical field, but transient and mild femoral neuropathy due to retracting the iliopsoas muscle and using radiofrequency (RF) devices occurred after surgery. It resolved after a few weeks of oral medication and the postoperative course was good, with improved mHHS, which reached 100. There were no remaining symptoms or recurrences at the two-year postoperative follow-up examination (Figure 7).

## Discussion

This study describes the successful treatment of iliopectineal bursitis associated with DDH using endoscopy and assesses the therapeutic effects, reduced invasiveness, and cosmetic outcomes, which may be especially important for young female patients. Iliopectineal bursitis is rarely primary, but often secondary to other hip diseases such as osteoarthritis, rheumatoid arthritis, synovial osteochondromatosis, and chronic inflammation associated with sporting activities [2,6]. No study has shown the direct relationship between bursitis and DDH. However, any condition able to generate a joint effusion and determining an elevation of intraarticular pressure may involve the iliopsoas bursitis [7]. Even in this case, it is considered that minimal labral injury associated with DDH may have increased the joint effusion and caused an expansion of the bursa. Conservative treatments, including rest, medications such as non-steroidal anti-inflammatory drugs, needle aspiration, and steroid injection, are effective first-line therapies. If conservative treatment fails or patients develop neuropathic or circulatory disorders due to stored fluid, surgery may be necessary [3-5]. Most previous studies have described open surgery, and the use of arthroscopy or endoscopy in these cases remains very limited.

This patient presented with recurrent iliopectineal bursitis with DDH and showed no signs of osteoarthritis, severe labral tearing, or any other hip disease, and was not regularly active in sports. These factors led us to suspect the existence of native communication between the intra-and extra-articular space with the one-way check-valve mechanism. Approximately 15% of typical individuals have communication between their bursa and hip joint [1]. In this case, the increased joint fluid caused by instability due to DDH and minimal labral injury appears to have accumulated in the extraarticular space through native communication, leading to symptomatic pain and a restricted range of motion. Consistent with this, the asymptomatic contralateral hip had mild fluid retention and communication between the intra- and extraarticular space.

In consideration of the invasion and cosmetic factors, endoscopic surgery was applied. We have previously performed endoscopy for tendinosis of the proximal head of the rectus femoris and obtained rapid pain relief and good results [8-10]. This procedure aimed to debride degenerated fat tissue around origin of the direct head of the rectus femoris muscle, decompress AIIS and release muscle adhesion. During surgery, we obtained a wide view of the anterior extraarticular hip joint space using only ALP and MAP portals. Operability can be further improved with additional portals, and this technique was applied in the present case. This method has several advantages. First, we can observe and treat various pathologies around hip joint, and if accompanying pathologies such as synovitis, tendinitis, and bony deformity are present, they can be treated simultaneously [8-11]. This patient also had minimal labral damage, which could cause joint fluid retention, and it was possible to repair it. However, her symptoms disappeared completely only by aspiration of fluid without injection for hip joint, and there were no clinical symptoms due to intra-articular pathologies such as anterior impingement sign, and she had DDH with a VCA angle of 13°; therefore, an intraarticular procedure was not performed. Second, endoscopy is minimally invasive compared with open surgery. Several studies have described the usefulness of minimally invasive endoscopic surgery for pathologies caused by extraarticular structures, such as the rectus femoris, iliopsoas, and abductor muscle [8-13]. Extraarticular endoscopy can be performed regardless of hip stability; therefore, it seems to contribute to the relief of various types of pain. Third, this method is cosmetically superior. Especially for a young woman, as in this case, endoscopy via just two small portals avoids skin incisions on the bikini line and is therefore preferred. However, because endoscopy requires more sophisticated techniques and clinician experience, it should be performed by well-trained surgeons. This patient has a postoperative transient femoral neuropathy that seems to be caused by retracting surrounding muscles and RF devices, and we must pay attention to surgical time and increasing temperature of the perfusate. The certainty of surgery can be secured by using intraoperative ultrasound and fluoroscopy.

This study has some limitations. First, bursectomy is not a radical treatment for this patient. Osteochondral degeneration due to labral tears and joint instability may progress in the future, and it cannot be denied that this method may be time-saving surgery up to curative surgeries such as osteotomy or arthroplasty. Second, we haven’t examined magnetic resonance arthrography that may have been able to evaluate more details of labrum damage and communication between intra- and extraarticular space.

## Conclusions

Recurrent and symptomatic iliopectineal bursitis sometimes requires surgical treatment. If there are no symptoms due to intraarticular pathologies, only bursa resection induces rapid pain relief even in the absence of labral repair. Endoscopic bursectomy for iliopectineal bursitis can be applied to patients with DDH, and effective, minimally invasive, cosmetically superior compared to open surgery, especially in young females.
